# Modelling the therapeutic dose range of single low dose primaquine to reduce malaria transmission through age-based dosing

**DOI:** 10.1186/s12879-017-2378-9

**Published:** 2017-04-08

**Authors:** Daniel Joseph Hayes, Clifford George Banda, Alexandra Chipasula-Teleka, Dianne Janette Terlouw

**Affiliations:** 1 Department of Clinical Sciences, Liverpool School of Tropical Medicine (LSTM), Liverpool, UK; 2 Malawi-Liverpool Wellcome Trust Clinical Research Programme, P.O. Box 30096, Chichiri Blantyre 3, Malawi

**Keywords:** Malaria, Primaquine, Dosing, Disease elimination, Transmission, Dose response relationship

## Abstract

**Background:**

Low-dose primaquine is a key candidate for use in malaria transmission reduction and elimination campaigns such as mass drug administration (MDA). Uncertainty about the therapeutic dose range (TDR) required for general and paediatric populations challenge the implementation of the World Health Organisation’s recommendation to add 0.25 mg/kg to current standard antimalarial treatment in such settings. Modelling work shows that for low-dose primaquine to have an impact, high efficacy and extensive population coverage are needed. In practice, age-based dose regimens, often used in MDA, could lead to safety concerns and a different effectiveness profile. We aimed to define TDRs for primaquine and to assess dosing accuracy of age-based dose regimens.

**Methods:**

Optimised regional age-based dosing regimens for low-dose primaquine were developed in steps. First, we identified potential TDR options based on suggested published efficacy and safety thresholds (i.e. 0.1–0.4, 0.125–0.375, 0.15–0.35 mg/kg). We then used our previously defined weight-for-age growth references and age-based dose optimisation tool to model predicted regimen accuracies for Africa, Asia and Latin America based on low-dose primaquine tablets (3.75 mg and 7.5 mg) currently under development by Sanofi while employing the identified dose range options and pre-specified regimen characteristics.

**Results:**

Dose regimens employing TDRs of 0.1–0.4 and 0.125–0.375 mg/kg had the highest average predicted dose accuracies in all regions with the widest dose range of 0.1–0.4 mg/kg resulting in ≥99% dose accuracy in all three regions. The narrower 0.15–0.35 mg/kg range was on average predicted to correctly dose 91.4% of the population in Africa, 93.2% in Asia and 92.6% in Latin America. This range would prescribe ≥20% of 3-year-olds doses below 0.15 mg/kg and ≥20% of 11-year-olds doses above 0.35 mg/kg. Widening the TDR from 0.15–0.35 to 0.1–0.4 mg/kg increased the dose accuracy by ≥20% in Africa, ≥15% in Asia and ≥10% in Latin America.

**Conclusion:**

Optimised age-based dose regimens derived from wider TDRs are predicted to attain the dose accuracies required for effective MDA in malaria transmission reduction and elimination settings. We highlight the need for a clearly defined TDR and for safety and efficacy trials to focus on doses compatible with age-based dosing often employed in such settings.

**Electronic supplementary material:**

The online version of this article (doi:10.1186/s12879-017-2378-9) contains supplementary material, which is available to authorized users.

## Background

Accurate dosing of antimalarials used in transmission-reduction strategies such as Mass drug administration (MDA) and Mass screening and treatment (MSaT) is just as important as accurate prescribing in the management of malaria patients. Underdosing with an antimalarial drug leads to low efficacy of the drug and renders it ineffective to clear parasites or kill gametocytes in the body and can be a catalyst for emergence of drug resistance. Overdosing predisposes individuals to toxicity and adverse events that may outweigh the individual benefits of taking the drug. This has the potential to have a significant impact on the success or failure of malaria control and elimination programs.

Dosing based on body weight is the standard in antimalarial drug development. However, population-based antimalarial mass treatment campaigns often dose individuals by their age rather than weight to avoid the need for weighing scales. While this practice can pose a serious threat to the targeted impact of mass treatment campaigns, the optimization and evaluation of the performance of age-based dose regimens has so far received relatively little attention. One of few studies that have looked at age-based dose regimen of other antimalarials in Africa, reported a high prevalence of systematic dosing errors (only 23.5% received the target dose of SP in children less than five years old) [1].

An antimalarial that comes highly recommended by WHO for use in reduction transmission is primaquine (PQ). It is licensed for the radical cure of *Plasmodium vivax* malaria and has also shown gametocytocidal activity in *P. falciparum*. In 2010, the WHO issued a recommendation to add a single dose of 0.75 mg/kg PQ to artemisinin-based combination therapies (ACTs) in mass treatment campaigns as a component of pre-elimination or elimination programmes [2]. Reports that followed indicated a high risk of haemolysis in glucose-6-phosphate dehydrogenase (G6PD) deficient individuals [3]. There was also documentation of haemolysis in non G6PD individuals who were co-administered PQ with ACT [4]. In 2012, the recommended target dose was reduced to 0.25 mg/kg and this removed the need for screening of G6PD deficiency [3]. The single low dose PQ (LDP) tested in G6PD individuals was slightly above and below the recommended 0.25 mg/kg (0.4 and 0.1 mg/kg) and no severe anaemia was observed [5]. To overcome the practical paediatric dosing challenges in children with the currently marketed 15 mg tablet with these altered recommendations, Sanofi and Medicines for Malaria Venture (MMV) are developing LDP tablets (7.5 mg and 3.75 mg) [6].

At present the WHO recommendation does not define a therapeutic dose range (TDR) for PQ and various dose optimization trials are still ongoing. A TDR is the interval between the minimum efficacious dose and the maximum tolerated dose in mg/kg body weight, based on the available evidence. This presents the acceptable variation in intake dose that informs the development of safe, accurate age- and weight-based dosing regimens. A wide TDR is better than a narrow TDR as it lowers the potential for over- and under-dosing [1, 7]. The question lies in how best the wide TDRs can be utilised to inform an age-based regimen that is both safe for the community and effective for the malaria control program. A clearly defined, wide TDR is particularly important for antimalarials used in mass treatment campaigns as part of transmission reduction efforts, such as MDA or MSaT. In these large scale population-based interventions user-friendly and logistically feasible, but at the same time accurate, dosing regimens are key. They are essential to achieve the required intervention coverage, drug exposure, patient adherence and dose accuracy across the population to impact transmission.

Accurate age-based dosing requires knowledge of the population weight-for-age (WFA) distribution across all ages and drugs with wide TDRs. We have previously developed tools to determine optimized age-based dose regimens using regional WFA growth references and drug characteristics including the TDR [8, 9]. We successfully applied them to help inform the drug ratio, tablet strength, and age-based dose regimen for the artesunate-amodiaquine fixed-dose combination (Coarsucam™) [10]. A large pooled patient level analysis of Coarsucam™ showed that the fixed dose combination provided higher efficacy in all age categories, which the investigators attributed to optimized dosing of artesunate-amodiaquine [11].

We therefore aimed to identify different potential TDRs for primaquine and to assess dosing accuracy of regional age-based dose regimens for the new tablet formulations using the tools described. We discuss how these findings can help inform the required TDR for primaquine and future dose optimization studies to maximize the health impact of mass treatment campaigns.

## Methods

We constructed optimised regional age-based dosing regimens for LDP for Africa, Asia and Latin America using the four steps detailed below. Optimised regimens were those age-based dose regimens with the highest predicted dose accuracy (the proportion of recipients who will be dosed within the assessed TDR) across all ages.

(i) Firstly, we conducted a literature review in September, 2015 in order to identify the TDRs for PQ from published data. We searched online electronic databases Medline, Embase and WHO search portal with no time restrictions using search terms “safety and efficacy” and “low dose primaquine”. The inclusion criteria were studies that had assessed safety and efficacy of PQ with defined TDRs. This search yielded a total of 3 studies all of which were included in the review [12–14].

(ii) Secondly, we identified three TDRs with different therapeutic indexes (TI) (TI = TDR upper threshold divided by the TDR lower threshold) and all are symmetrically placed around the WHO recommended target dose of 0.25 mg/kg. These are (i) 0.15–0.35 mg/kg, with a narrow TI of 2.3. (ii) 0.125–0.375 mg/kg with a TI of 3. (iii) 0.1–0.4 mg/kg with a wider TI of 4. Our lowest cut off point was 0.125 mg/kg which has been shown to achieve maximal gametocytocidal efficacy within 24 to 48 h of administration in adults [12, 14]. Our highest cut off point was 0.4 mg/kg. Eziefula and colleagues reported that 0.4 mg/kg had a gametocytocidal efficacy similar to 0.75 mg/kg (mean duration of gametocyte carriage: 6.6 days in the 0.75 mg/kg group, and 6.3 days in the 0.4 mg/kg group) [13]. Doses of PQ above 0.5 mg/kg have been associated with serious adverse events [12].

(iii) Thirdly, we developed regional WFA growth references for malaria endemic regions, details of which are described elsewhere [8]. Briefly, a database of over 1 million weight-for-ages from population representative surveys in over 64 malaria endemic countries was compiled. We employed the previously developed extension to the standard Generalised Additive Model for Location, Scale and Shape (GAMLSS) to model data from multiple sources and varying growth distributions [9]. To increase their applicability for the optimisation of age-based dosing of antimalarials the references were weighted for population size at risk of falciparum or vivax malaria [15], and integrated into a previously developed age-based regimen optimisation tool [10].

(iv) Lastly, we employed these references and modelled our three TDRs to assess the impact that the width of a therapeutic range has on the predicted dose accuracy of a regimen. We calculated the percentage of the population predicted to receive a PQ intake dose over, under and within the TDR using a dose optimisation tool developed within our team [10]. Predicted dose accuracies were generated by age for a given TDR, tablet strength and the number of dose categories in a regimen.

In our analysis we used the following restrictions (Additional file 1: Table S1): (i) The regimen had to be a single dose regimen. (ii) The regimen had to be based on only two tablet strengths, i.e. a 3.75 mg ‘paediatric’ (p) and a 7.5 mg ‘adult’ (a) tablet. (iii) The regimen could contain only dose categories of 0.5p, 1p, 1a, and 1a + 1p. The age range was 6 months to 50 years. We set the upper and lower therapeutic range cut-offs to 0.025 mg/kg increments. The use of tablet fractions was only allowed for the smallest administrable dose to ensure simple user friendly regimens appropriate for mass treatment campaigns (note that due to the size of the tablets, the new tablets may not be scored). We assumed a linear dose response in all age groups.

## Results

All age-based dose regimen cut-offs and their dosing accuracies are presented in Table 1 below. Average dosing accuracies for the regional optimised regimens using a dose range of 0.15–0.35 mg/kg were: 91.4% for Africa, 93.2% for Asia and 92.6% for Latin America. The wider 0.1–0.4 mg/kg dose range had the highest average dosing accuracies in all regions as follows: 99.6% for both Africa and Asia, 99.9% for Latin America.

**Table 1:** Performance of optimized PQ regimen options by region and assessed TDRs

TDR:0.125–0.375 mg/kg	Africa		Asia		Latin America	
Dose	Age	Accuracy	Age	Accuracy	Age	Accuracy
(tablets)	(yrs)	(%)	(yrs)	(%)	(yrs)	(%)
0.5p	6mo-3	97.9	6mo-4	96.1	6mo-3	97.1
1p	4–10	97.5	5–12	97.6	4–8	98.5
1a	11–16	96.4	13–16	97.7	9–13	97
1a + 1p	17+	99.6	17+	99.8	14+	99.1
		97.9		97.8		97.9
TDR:0.15–0.35 mg/kg	Africa		Asia		Latin America	
Dose	Age	Accuracy	Age	Accuracy	Age	Accuracy
(tablets)	(yrs)	(%)	(yrs)	(%)	(yrs)	(%)
0.5p	6mo-3	88.2	6mo-3	92	6mo-2	94.8
1p	4–10	90.3	4–11	92.9	3–8	90.3
1a	11–15	91.4	12–16	89.3	9–13	91.1
1a + 1p	16+	95.8	17+	98.6	14+	94
		91.4		93.2		92.6
TDR:0.1–0.4 mg/kg	Africa		Asia		Latin America	
Dose	Age	Accuracy	Age	Accuracy	Age	Accuracy
(tablets)	(yrs)	(%)	(yrs)	(%)	(yrs)	(%)
0.5p	6mo-4	99.3	6mo-4	99.4	6mo-3	100
1p	5–11	99.7	5–12	99.6	4–9	99.6
1a	12–16	99.3	13–19	99.5	10–14	99.8
1a + 1p	17+	100	20+	100	15+	100
		99.6		99.6		99.9

0.5p = 1.875, 1p = 3.75 mg, 1a = 7.5 mg, 1a + 1p = 11.25 mg, *PQ* Primaquine, *MO* months. *TDR* Therapeutic Dose Range

**Fig. 1:**
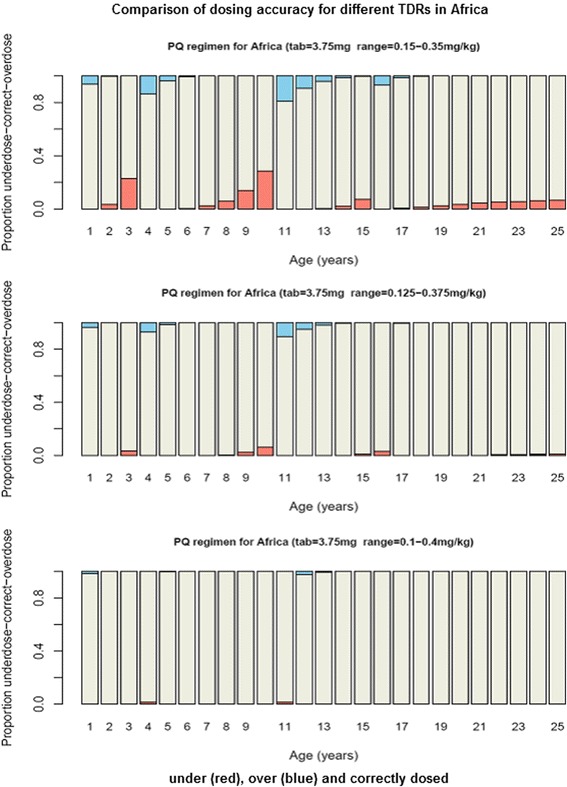
Comparison of dosing accuracy for modelled dose ranges in Africa. Population under (red), over (blue) and correctly (white) dosed by age for Africa using three dose ranges (0.1–0.4, 0.125–0.375 and 0.15–0.35 mg/kg). PQ = Primaquine, TDR = Therapeutic dose range, tab = tablet strength

**Fig. 2:**
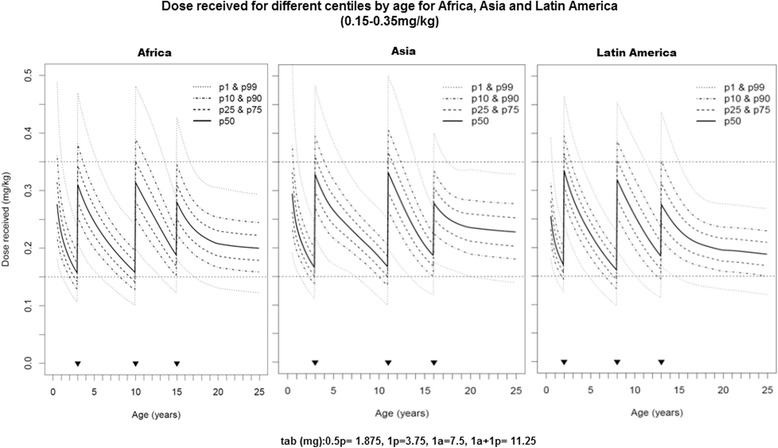
Predicted intake dose for selected body weight centiles by age for Africa, Asia and Latin America using a dose range of 0.15–0.35 mg/kg. Proportion of the population in the three regions predicted to receive doses between 0 and 15-0.35 mg/kg (tab = 3.75 mg) by age for PQ, black triangle indicates regimen cut-offs on the horizontal axis as follows: Africa = 6 months (mo)-3, 4–10, 11–15 and 16+ years; Asia =6mo-3, 4–11, 12–16 and 17+ years; Latin America = 6mo-2, 3–8, 9–13 and 14+ years

The modelled optimized regimen with a dose range of 0.15–0.35 mg/kg in Africa resulted in underdosing among 3-year-olds with more than 20% receiving doses below 0.15 mg/kg (Fig. 1). A similar proportion of 11-year-olds would be overdosed (Fig. 1), receiving more than 0.35 mg/kg, though most would receive a dose between 0.125–0.375 mg/kg (Fig. 2). The highest proportion of underdosing in Asia would be seen in the 11-year-olds (close to 18% of children) while 12-year-olds would be overdosed in >20% of cases (Additional file 2: Figure S1). In Latin America, ≥20% of 3- and 8-year-olds would be over- and underdosed respectively (Additional file 3: Figure S2).

TDRs of 0.125–0.375 mg/kg and 0.1–0.4 mg/kg resulted in higher dosing accuracies than the 0.15–0.35 mg/kg range (Table 1 and Additional file 4: Figure S3). On further analysis, widening the lower and upper cut-offs of the TDR from 0.15 to 0.1 mg/kg and 0.35 to 0.4 mg/kg respectively, increased the dosing accuracy by ≥20% in Africa, ≥15% in Asia, ≥10% in Latin America, with the highest accuracy levels of ≥97% seen with the widest range of 0.1–0.4 mg/kg across all age groups in the three regions.

## Discussion

The need for antimalarial dose optimization to focus on intervention delivery and end users is increasingly recognized. This is even more important for gametocytocidal drugs like PQ, where no direct benefit to the individual taking the drug exists, and the risk-benefit argument is shifted from individual to population level. Minimising the risk of dose related adverse events in potentially healthy individuals across the population, while achieving the high population coverage and high efficacy across vulnerable subgroups that drives the health impact of the intervention will be strongly dependent on the implemented dose regimens.

We show that optimised age-based regional regimens would achieve accurate dosing within the assessed TDRs in over 90% of the population (Table 1, Fig. 1, Additional file 2: Figure S1, Additional file 3: Figure S2 and Additional file 4: Figure S3). The narrow TDR of 0.15–0.35 mg/kg (a TI of 2.3) however may not be acceptable in transmission reduction and elimination treatment campaigns as a large proportion of children in age categories bordering dose cut-offs would not get a dose within the TDR (Fig. 2). With a TDR of 0.1–0.4 mg/kg (a TI of 4) >99% of the populations in the 3 regions could be accurately dosed. As defining the TDR for LDP is still on-going, our findings can help design future field trials and inform discussions on the needed TDR and TI in several ways. They highlight the benefit of optimizing age-based regimens in contributing to dose accuracy and likely health impact of mass treatment campaigns. They help identify the target TDR (and TI) that needs to be assessed and confirmed in field trials to dose ideally 100% of a population within that range. And finally, they demonstrate the importance for TDRs to be symmetrically placed around the target dose for drugs that will be widely dosed by age, a requirement that is frequently overlooked. Our modelling methods are complementary to the more standardized pharmacokinetic-pharmacodynamic (PK-PD) modelling techniques to assess the minimum and maximum TDR thresholds in vulnerable subgroups as promoted by the Worldwide Antimalarial Resistance Network (WWARN) [11].

In our models, we included the vulnerable subgroup of children. We recognise that this subgroup does not always follow a linear dose response due to differences in pharmacokinetics within the group. The PK-PD profile of this group can therefore follow a different pattern from that shown in adults. However, an assumption of a linear dose response across all age groups including children was made in this analysis, in view of the lack of evidence of PK-PD models of LDP in paediatric settings. Further research is needed on this aspect.

In case-management of uncomplicated malaria the agreed minimum acceptable level of efficacy of an antimalarial is 90% [16]. While this is applied to manufacturer regimens, WHO recommended weight-based regimens, and other bespoke weight-based trial regimens, age-based regimens are rarely assessed for their efficacy, and national control programmes can implement various age-based dose recommendations without ever assessing their efficacy. Similarly, there is no consensus on the minimum expected level of either drug efficacy or dosing accuracy for transmission reduction in malaria elimination settings. This lack of guidance hampers the establishment of suitable dose regimens for MDA campaigns employed in such settings. Age-based regimens with overall dose accuracy over 90% that significantly over- and underdose particular age groups can be evaluated on their suitability and effectiveness to control transmission in targeted efficacy studies (e.g. by assessing the efficacy in those specific age and weight subgroups predicted to receive doses below the TDR) before they are assessed in large scale population trials. The use of targeted safety studies and pharmacovigilance in subgroups who receive intake doses above the upper TDR threshold (particularly individuals in the age categories bordering dose cut-offs) could also help accumulate key safety data in a short-period and help define practical regimens and acceptable dosing levels for populations.

By visualizing the loss in dosing precision with age-based dosing at population level, we call for a more careful review by policy makers and control programme managers to balance the risks and benefits of age-based versus weight-based dosing in programmes directed at reducing malaria transmission. While weight-based doses pose considerable logistical challenges due to the need for and maintenance of large numbers of weighing scales, these should be carefully reviewed against any predicted or unknown health impact and safety concerns with the identified TDRs and associated age-based dosing regimens.

One of the main strengths of our study is that we have utilised the tablet sizes and strengths that are already established on the market which makes it easier for future studies to implement. Our study explores the dose range that can be used in relation to the tablet size and strength. However, the concept is not limited to the tablets used in this study. It can be applied to other future tablet sizes and strengths should they be developed. Another strength lies in the fact that the age-based dose regimen of each region reflects its population as it is based on each region’s weight for age growth references.

Our model is limited in that it does not take into account how the safety margins of LDP may shift in G6PD deficient individuals. Currently, there are no guidelines of how to adapt the dosing in the variants of G6PD deficiency, especially taking into account that PQ causes a dose-dependent haemolysis [17]. Wickham et al., reports that LDP that was shown to be safe in African G6PD deficient individuals was shown to induce haemolysis in Mediterranean variant of G6PD in a mouse model [18]. Therefore, the difficulty will be in determining the lower safety margin and risk of underdosing in the African variant of G6PD. On the other hand, the upper safety margin and the risk of overdosing in Mediterranean variant of G6PD will need to be established. Further studies on the safety margins of LDP in G6PD deficient individuals will need to be conducted in order to get an accurate picture.

## Conclusions

In conclusion, our findings highlight the potential impact on the expected dose accuracy with shift in the agreed TDR and the need for clearly established minimum width of the TDR through safety and efficacy studies. This needs to be assessed with the feasibility of age-based dosing of single low dose PQ to reduce transmission of malaria. This principle is further relevant for any drug with an age-based dose regimen.

## Additional files

Additional file 1: Table S1. Modelled regimen characteristics. Summary of the regimen characteristics used in the models. (DOCX 11 kb)

Additional file 2: Figure S1. Comparison of dosing accuracy for different therapeutic dose ranges in Asia. Population under (red), over (blue) and correctly (white) dosed by age for Africa using three dose ranges (0.1–0.4, 0.125–0.375 and 0.15–0.35 mg/kg). PQ = Primaquine, TDR = Therapeutic dose range, tab = tablet strength. (TIFF 117 kb)

Additional file 3: Figure S2. Comparison of accuracy for different therapeutic dose ranges in Latin America. Population under (red), over (blue) and correctly (white) dosed by age for Africa using three dose ranges (0.1–0.4, 0.125–0.375 and 0.15–0.35 mg/kg). PQ = Primaquine, TDR = Therapeutic dose range, tab = tablet strength. (TIFF 130 kb)

Additional file 4: Figure S3. Predicted dose intake for selected body weight centiles by age for Africa, Asia and Latin America. Proportion of the population in the three regions predicted to receive doses between 0.1–0.4 mg/kg (plane dotted line) and 0.125–0.375 mg/kg (bold dotted line) by age for Primaquine, black triangle indicates regimen cut-offs on the horizontal which for the 0.1–0.4 mg/kg range translated to the following regimen cut offs: Africa = 6 months (mo) -4, 5–11, 12–16 and 17+ years; Asia =6mo-4, 5–12, 13–19 and 20+ years; Latin America = 6mo-3, 4–9, 10–14 and 15+ years. A similar illustration for the 0.125–0.375 mg/kg resulted in the following regimen cut-offs: Africa = 6 months (mo) -3, 4–10, 11–16 and 17+ years; Asia =6mo-4, 5–12, 13–16 and 17+ years; Latin America = 6mo-3, 4–8, 9–13 and 14+ years. (TIFF 120 kb)

## Abbreviations

ACTs: Artemisinin-based combination therapies
G6PD: Glucose-6-phosphate dehydrogenase
GAMLSS: Generalised Additive Model for Location, Scale and Shape
LDP: Low-dose primaquine tablets
MDA: Mass drug administration
MMV: Medicines for malaria venture
MSaT: Mass screening and treatment
PK-PD: Pharmacokinetic-pharmacodynamic
PQ: Primaquine
TDR: Therapeutic Dose Range
WFA: Weight-for-age
WHO: World Heath Organisation
