# Supplementary material for: Modelling the therapeutic dose range of single low dose primaquine to reduce malaria transmission through age-based dosing
Source: BMC Infect Dis. 2017 Apr 8;17:254. doi: 10.1186/s12879-017-2378-9 (PMC5385020; doi:10.1186/s12879-017-2378-9)
Supplement: Supplementary file 1 — Modelled regimen characteristics. Summary of the regimen characteristics used in the models. (DOCX 11 kb) [file 12879_2017_2378_MOESM1_ESM.docx]

| **Additional file 1: Table S1: Modelled regimen characteristics** | |
| --- | --- |
| Modelled Parameters | Input Values |
| Dosing schedule | Single dose |
| Lower cut-offs | 0.1, 0.125, 0.15 mg/kg |
| Upper cut-offs | 0.35, 0.375, 0.4mg/kg |
| Number of dose categories | 4 |
| Tablet strengths | 3.75mg, 7.5mg |
| Tablet options | 0.5p, 1p, 1a, and 1a + 1p |
| Over vs. under-dosing | Equal importance |
| Age-range | 6months -50yrs |
| Target population | African, Asian and Latin America |
