# Supplementary figures and images for: Modelling the therapeutic dose range of single low dose primaquine to reduce malaria transmission through age-based dosing
Source: BMC Infect Dis. 2017 Apr 8;17:254. doi: 10.1186/s12879-017-2378-9 (PMC5385020; doi:10.1186/s12879-017-2378-9)

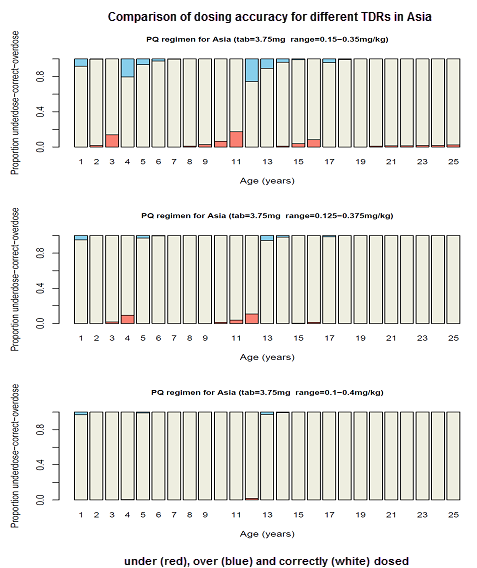

Supplement: Supplementary file 2 — Comparison of dosing accuracy for different therapeutic dose ranges in Asia. Population under (red), over (blue) and correctly (white) dosed by age for Africa using three dose ranges (0.1–0.4, 0.125–0.375 and 0.15–0.35 mg/kg). PQ = Primaquine, TDR = Therapeutic dose range, tab = tablet strength. (TIFF 117 kb) [file 12879_2017_2378_MOESM2_ESM.tif]

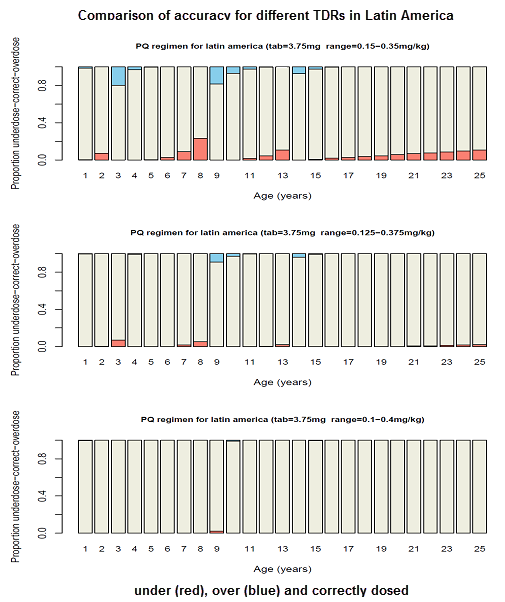

Supplement: Supplementary file 3 — Comparison of accuracy for different therapeutic dose ranges in Latin America. Population under (red), over (blue) and correctly (white) dosed by age for Africa using three dose ranges (0.1–0.4, 0.125–0.375 and 0.15–0.35 mg/kg). PQ = Primaquine, TDR = Therapeutic dose range, tab = tablet strength. (TIFF 130 kb) [file 12879_2017_2378_MOESM3_ESM.tif]

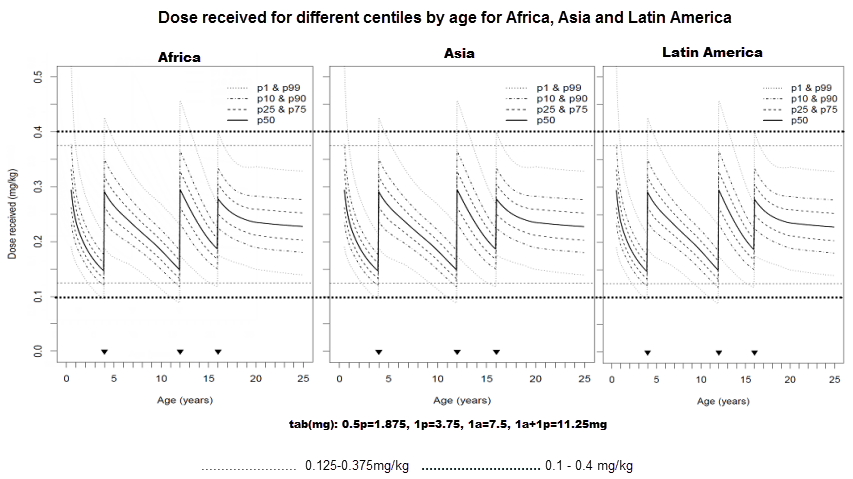

Supplement: Supplementary file 4 — Predicted dose intake for selected body weight centiles by age for Africa, Asia and Latin America. Proportion of the population in the three regions predicted to receive doses between 0.1–0.4 mg/kg (plane dotted line) and 0.125–0.375 mg/kg (bold dotted line) by age for Primaquine, black triangle indicates regimen cut-offs on the horizontal which for the 0.1–0.4 mg/kg range translated to the following regimen cut offs: Africa = 6 months (mo) -4, 5–11, 12–16 and 17+ years; Asia =6mo-4, 5–12, 13–19 and 20+ years; Latin America = 6mo-3, 4–9, 10–14 and 15+ years. A similar illustration for the 0.125–0.375 mg/kg resulted in the following regimen cut-offs: Africa = 6 months (mo) -3, 4–10, 11–16 and 17+ years; Asia =6mo-4, 5–12, 13–16 and 17+ years; Latin America = 6mo-3, 4–8, 9–13 and 14+ years. (TIFF 120 kb) [file 12879_2017_2378_MOESM4_ESM.tif]
